# Respiration of metal (hydr)oxides by *Shewanella* and *Geobacter*: a key role for multihaem *c*-type cytochromes

**DOI:** 10.1111/j.1365-2958.2007.05783.x

**Published:** 2007-07-01

**Authors:** Liang Shi, Thomas C Squier, John M Zachara, James K Fredrickson

**Affiliations:** Pacific Northwest National Laboratory Richland, Washington 99354, USA.

## Abstract

Dissimilatory reduction of metal (e.g. Fe, Mn) (hydr)oxides represents a challenge for microorganisms, as their cell envelopes are impermeable to metal (hydr)oxides that are poorly soluble in water. To overcome this physical barrier, the Gram-negative bacteria *Shewanella oneidensis* MR-1 and *Geobacter sulfurreducens* have developed electron transfer (ET) strategies that require multihaem *c*-type cytochromes (*c*-Cyts). In *S. oneidensis* MR-1, multihaem *c*-Cyts CymA and MtrA are believed to transfer electrons from the inner membrane quinone/quinol pool through the periplasm to the outer membrane. The type II secretion system of *S. oneidensis* MR-1 has been implicated in the reduction of metal (hydr)oxides, most likely by translocating decahaem *c*-Cyts MtrC and OmcA across outer membrane to the surface of bacterial cells where they form a protein complex. The extracellular MtrC and OmcA can directly reduce solid metal (hydr)oxides. Likewise, outer membrane multihaem *c*-Cyts OmcE and OmcS of *G. sulfurreducens* are suggested to transfer electrons from outer membrane to type IV pili that are hypothesized to relay the electrons to solid metal (hydr)oxides. Thus, multihaem *c*-Cyts play critical roles in *S. oneidensis* MR-1- and *G. sulfurreducens*-mediated dissimilatory reduction of solid metal (hydr)oxides by facilitating ET across the bacterial cell envelope.

## Introduction

*c*-Type cytochromes (*c*-Cyts) are ubiquitous in nearly all living organisms, where they play vital roles in mediating electron transfer (ET) reactions associated with respiration. Although their amino acid sequences differ greatly, all *c*-Cyts possess at least one haem that is covalently bound through amino acid side-chains of the proteins to position and orient the haem moiety and thereby facilitate efficient reactions. The haem moieties are commonly co-ordinated through two thioester bonds to proximal cysteines in the protein, where the signature motif of most *c*-Cyts is CX_2_CH (other common motifs include CX_3−4_CH, CX_2_CK and A/FX_2_CH). These motifs with covalently bound haems are the key components used to constitute the haem-containing domains whose diverse functions range from binding of O_2_ and catalysis to electron transfer and accumulation (Stevens *et al.*, 2004; Rodrigues *et al*., 2006). *c*-Cyts have been extensively investigated, and several excellent reviews have been dedicated to the structures, chemistry and biogenesis of *c*-Cyts (Stevens *et al.*, 2004; Mowat and Chapman, 2005). This review focuses on the unique features of bacterial *c*-Cyts with multiple haems and their roles in bacteria-mediated dissimilatory reduction of solid metal (hydr)oxides.

## The unique features of bacterial *c*-Cyts with multiple haems

During the last decade, high-throughput DNA sequencing of bacterial genomes has revealed that some facultatively or strictly anaerobic bacteria, such as the dissimilatory metal-reducing bacteria (DMRB) *Shewanella oneidensis* MR-1 and *Geobacter sulfurreducens*, contain numerous *c*-Cyts (predicted 42 and 111, respectively, compared with only 7 in *Escherichia coli*) (Heidelberg *et al*., 2002; Methe *et al*., 2003). The *c*-Cyts are essential for the versatile anaerobic respiration capabilities of *S. oneidensis* MR-1 because mutants defective in the *c*-Cyt maturation system are unable to produce functional *c*-Cyts and consequently fail to grow when fumarate, dimethyl sulphoxide (DMSO) or trimethylamine *N*-oxide (TMAO) is used as the terminal electron acceptor (Bouhenni *et al*., 2005). Genome sequence analysis has also revealed that most of these *c*-Cyts polypeptides found in DMRB possess more than one CX_2_CH motif, and that one of these putative *c*-Cyts in *G. sulfurreducens* has as many as 27 CX_2_CH motifs, in sharp contrast to the *c*-Cyts found in eukaryotes, which typically contain only one haem (Heidelberg *et al*., 2002; Methe *et al*., 2003). Some multihaem *c*-Cyts found in DMRB are located in the outer membrane, where they are positioned to interact with extracellular substrates, whereas most membrane *c*-Cyts found in other bacteria, including multihaem *c*-Cyts in sulphate respiring bacteria such as *Desulfovibrio*, are associated with the cytoplasmic or inner membranes (Heidelberg *et al*., 2004).

Although their overall three-dimensional (3-D) structures vary considerably, one of the unique features found in most bacterial multihaem *c*-Cyts whose 3-D structures have been solved is the arrangement of haem groups. In these multihaem *c*-Cyts, all haem groups are positioned in such way that each is in close proximity to at least one of the other haems, and the porphyrin rings of two adjacent haems are positioned either parallel or perpendicular to each other. These arrangements are thought to facilitate rapid ET with considerable specificity among the haem groups that form a continuous ‘electric wire’ (Mowat and Chapman, 2005). When protein complexes are formed among multihaem *c*-Cyts, at least one haem group in one *c*-Cyt subunit is usually positioned close to a haem group in another *c*-Cyt subunit, again permitting rapid and specific inter-ET between the proximal subunits (Rodrigues *et al*., 2006). Formation of protein complexes among multihaem *c*-Cyts and the close arrangement of haem groups within and between multihaem *c*-Cyts make it possible to transfer electrons rapidly over relatively long distances. The *c*-Cyts quinol dehydrogenase (NrfH)/nitrite reductase (NrfA) complex of *Desulfovibrio vulgaris* consists of two NrfHs and four NrfAs with a total of 28 haems that are used to form the entire ET network of the NrfH/NrfA complex. The longest distance that electrons could flow from the haem possibly used for quinol oxidation in one of the NrfH subunits to the haem for nitrite reduction in an NrfA subunit along the haem network (centre-to-centre) is ∼98 Å (or 9.8 nm), in which 10 haems are involved (Cunha *et al*., 2003; Rodrigues *et al*., 2006).

In addition to their role in ET, some of the bacterial *c*-Cyts with multiple haems are believed to function as capacitors to accumulate electrons (Rodrigues *et al*., 2006). One of the possible reasons to accumulate electrons is that some of the chemical reactions catalysed by these *c*-Cyts require multiple electrons (e.g. six-electron reduction of nitrite to ammonia by NrfA). Accumulation of a sufficient number of electrons might help complete the chemical reactions (Rodrigues *et al*., 2006). Thus, the ability to pack multiple haem groups densely in *c*-Cyts through their covalent attachment to the protein backbone, as opposed to the more frequently found Cyts with non-covalently bound haem (e.g. *b*-type Cyts) (Stevens *et al*., 2004), not only facilitates efficient ET over a relatively long distance, but might also be well suited for efficient accumulation of electrons for catalysing specific chemical reactions.

## Dissimilatory reduction of solid metal (hydr)oxides: a challenge for electron transfer by microbial cells

The ability of bacteria to use oxidized metals, such as iron [Fe(III)] or manganese [Mn(III, IV)] (hydr)oxides, as terminal electron acceptors to generate energy for biosynthesis and cell maintenance (i.e. dissimilatory metal reduction), was originally discovered in the Gram-negative bacteria *S. oneidensis* MR-1 and *Geobacter metallireducens* nearly two decades ago (Lovley and Phillips, 1988; Myers and Nealson, 1988). Since then, the dissimilatory metal reduction activity has been observed not only in other Gram-negative bacteria, but also in Gram-positive bacteria and Archaea (Lovley, 2006; Weber *et al*., 2006). This metal reduction activity plays an important role in: (i) global carbon and nutrient cycling, (ii) the weathering and formation of minerals, (iii) the remediation of metal contaminants such as uranium (U), technetium and chromium, and (iv) harvesting electricity generated from bacterial-mediated biomass conversion involving waste treatment (Gorby *et al*., 2006; Lovley, 2006; Marshall *et al*., 2006; Xiong *et al*., 2006).

Reduction of Fe(III)/Mn(III, IV) (hydr)oxides, however, requires specific mechanisms to overcome physical limitations associated with ET to the (hydr)oxides because: (i) Fe(III)/Mn(III, IV) (hydr)oxides are poorly soluble in water at neutral pH and in the absence of strong complexing ligands [e.g. 10^−18^ M for Fe(III)] (Schröder *et al*., 2003), and (ii) nearly all DMRB contain cell walls and outer membranes in the case of the Gram-negative bacteria that physically separate cytoplasmic membranes, where electrons are usually accumulated, from insoluble and extracellular Fe(III)/Mn(III, IV) (hydr)oxides. The distance that electrons need to cross from the inner membrane to the bacterial cell surface is at least 8 nm (Prescott *et al*., 1996), too great a distance for electrons to cross via a tunnelling mechanism that is limited to < 1.4 nm (Leys and Scrutton, 2004). To overcome these physical obstacles, several ET strategies have evolved among different DMRB, including (i) physical transfer of electrons from the cytoplasmic membrane across the cell envelope to the surface of solid metal (hydr)oxides external to the cell, (ii) production of organic complexing ligands such as siderophores that are excreted to the extracellular milieu to solubilize the metal (hydr)oxides (Weber *et al*., 2006), and (iii) production of low-molecular-weight redox-active organic molecules that shuttle electrons between oxidized and reduced compounds (Hernandez and Newman, 2001). Although detailed information on the underlying mechanisms are lacking, bacterial multihaem *c*-Cyts are known to be the principal components directly involved in facilitating ET from the inner membrane to the surface of solid metal (hydr)oxides.

**Fig. 1:**
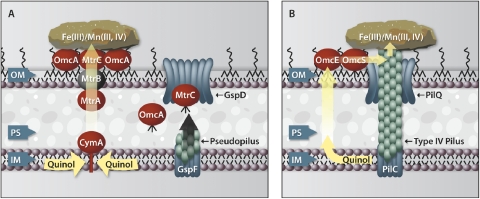
Proposed models depicting electron transfer pathways for *S. oneidensis* MR-1 (A) and *G. sulfurreducens* (B) during dissimilatory reduction of solid metal (hydr)oxides. For simplicity, the quinone-reducing portion of respiratory chain, the peptidoglycan layer and the individual components of the type II secretion system (T2S) and type IV pilus (T4P) biogenesis machine (other than GspD/PilQ, GspF/PilC and pseudopilus/pilus apparatus) are omitted from these models. Identified multihaem *c*-type cytochromes (*c*-Cyts) are in red. Yellow arrows indicate the proposed electron transfer (ET) path. A. As a member of NapC/NirT family of quinol dehydrogenases, inner membrane (IM) *c*-Cyt CymA of *S. oneidensis* MR-1 is capable of oxidizing quinol at IM and reducing the redox proteins, such as *c*-Cyt MtrA, at periplasm (PS). MtrA might also interact with the outer membrane (OM) protein MtrB. Although it is not a *c*-Cyt, MtrB is speculated to facilitate ET across OM to MtrC, an OM *c*-Cyt. Pseudopilus apparatus of T2S, whose formation is regulated by a protein complex in the IM, where only GspF is shown, pushes MtrC and OmcA (another OM *c*-Cyt) from PS through GspD to the surface of bacterial cells where MtrC and OmcA form a functional complex. The cell surface MtrC and OmcA are capable of directly reducing solid Fe(III)/Mn(III, IV) (hydr)oxides. B. In *G. sulfurreducens*, OM *c*-Cyts OmcE and OmcS are suggested to transfer electrons to the T4P apparatus, which then transfers electrons directly to solid Fe(III)/Mn(III, IV) (hydr)oxides. The structural components that mediate ET from the IM to OmcE/OmcS in the OM during reduction of solid metal (hydr)oxides have yet to be identified experimentally.

Considerable work has been done in *S. oneidensis* MR-1, where a range of different multihaem *c*-Cyts provide the structural and electrochemical means to mediate ET from the quinone/quinol pool of the inner membrane, to the periplasm, to the outer membrane and finally to the Fe(III)/Mn(III, IV) (hydr)oxides outside the cell (Fig. 1A). This conceptual model differs considerably from that proposed for *G. sulfurreducens*, which has been suggested to transfer electrons from *c*-Cyts in the outer membrane to solid metal (hydr)oxides through an elongated type IV pili (T4P), or geopili, that is electrically conductive (Reguera *et al*., 2005; Lovley, 2006) (Fig. 1B). The latter model, however, currently lacks a mechanistic explanation for how electrons are transferred via pilin proteins.

Because of their critical roles in ET to solid metal (hydr)oxides in *S. oneidensis* MR-1, we will first discuss in detail the current knowledge regarding the multihaem *c*-Cyts of *S. oneidensis* MR-1, which are directly involved in the ET to solid metal (hydr)oxides. We will then discuss the possible roles of multihaem *c*-Cyts of *G. sulfurreducens* in ET to solid metal (hydr)oxides and current evidence suggesting functional similarities and differences in the reduction of extracellular metal (hydr)oxides by these DMRB.

## The multihaem *c*-Cyts directly involved in *S. oneidensis* MR-1-mediated dissimilatory reduction of solid metal (hydr)oxides

### CymA: an inner membrane *c*-Cyt that serves as an entry point for electron transfer from the inner membrane quinone/quinol pool to the periplasm

CymA (SO_4591) of *S. oneidensis* MR-1 is a tetrahaem *c*-Cyt that shares some sequence similarity to the members of NapC/NirT family of quinol dehydrogenases (Table 1). Like other members of the NapC/NirT family, CymA contains a short N-terminal region that is anchored in the inner membrane and a long C-terminal region that binds four haems and protrudes into the periplasm (Fig. 1A). Deletion of the gene encoding CymA significantly impedes the ability of *S. oneidensis* MR-1 (i.e. 80–100%) to use a range of substrates as terminal electron acceptors, including Fe(III)/Mn(IV) oxides, fumarate, nitrate, nitrite and DMSO (Myers and Myers, 1997; 2000; Schwalb *et al*., 2003; Lies *et al*., 2005). The residual Fe(III)/Mn(IV) oxides reduction activity of *cymA*-inactivation mutant indicates that other proteins whose function is similar to that of CymA might also be involved in reduction of solid metal oxides.

**Table 1:** The multihaem *c*-type cytochromes directly involved in *Shewanella oneidensis* MR-1- or *Geobacter sulfurreducens*-mediated reduction of solid Fe(III)/Mn(IV) oxides.

Species	Cyt/locus tag	No. of haems	MM (kDa)	Location
*Shewanella oneidensis* MR-1	CymA/SO_4591	4	20	PS side of IM
	MtrA/SO_1777	10^a^	36	PS
	MtrC/SO_1778	10^a^	71	ES side of OM
	OmcA/SO_1779	10^a^	78	ES side of OM
*Geobacter sulfurreducens*	OmcE/GSU0618	4	27	ES side of OM^b^
	OmcS/GSU2504	6	51	ES side of OM^b^
	OmcT/GSU2503	6	45	Unknown

a The numbers of haem group per polypeptide of these Cyts were determined experimentally (Pitts *et al*., 2003; Shi *et al*., 2006).

b Their locations need to be validated in future experiments.

Cyt, cytochrome; MM, molecular mass; PS, periplasm; IM, inner membrane or cytoplasmic membrane; ES, extracellular space; OM, outer membrane.

CymA is believed to serve as an ET intermediate between the quinone/quinol pool of the inner membrane and the terminal reductases for fumarate, nitrate and nitrite, which are located in the periplasm, as well as the periplasmic proteins involved in delivering electrons across periplasm to the outer membrane to reduce solid Fe(III)/Mn(IV) oxides and DMSO (Gralnick *et al*., 2006). Consistent with this periplasmic function, truncated CymA lacking an N-terminal membrane-anchoring domain retains the ability to transfer electrons directly to FccA (formerly known as Fcc_3_), the respiratory fumarate reductase of *S. oneidensis* MR-1, with a second-order rate constant of 19 μM^−1^ s^−1^ (Maier *et al*., 2003; Schwalb *et al*., 2003). In contrast to FccA, no ET is observed from CymA to cytochrome *c*_3_, a periplasmic tetrahaem *c*-Cyt of *S. oneidensis* MR-1, demonstrating that the ET from CymA to FccA is specific (Schwalb *et al*., 2003). In some members of ε- and δ-proteobacteria, such as *Wolinella* and *Desulfovibrio*, NrfH, a CymA homologue, forms a stable complex with NrfA (Rodrigues *et al*., 2006). Given that deletion of *cymA* abolishes the ability of *S. oneidensis* MR-1 to reduce nitrite (Schwalb *et al*., 2003) and that CymA is a homologue of NrfH, CymA is believed to interact directly with NrfA (SO_3980) of *S. oneidensis* MR-1.

### MtrA: a periplasmic *c*-Cyt that transfers electron across the periplasm?

The gene encoding MtrA (SO_1777) is located within a four-gene cluster that also includes genes encoding three outer membrane proteins: MtrB (SO_1776), MtrC or OmcB (SO_1778) and OmcA (SO_1779). All have been linked to dissimilatory reduction of insoluble forms of Fe(III) and/or Mn(IV) oxides (Beliaev and Saffarini, 1998; Beliaev *et al*., 2001; Myers and Myers, 2001; 2002a; Lies *et al*., 2005; Gorby *et al*., 2006). MtrA is a soluble *c*-Cyt with 10 haems (Table 1) that is localized to the periplasm (Pitts *et al*., 2003). Compared with wild-type (wt) *S. oneidensis* MR-1, a mutant lacking MtrA exhibits attenuated reduction of Fe(III)-citrate and MnO_2_, while its ability to reduce fumarate, nitrate, nitrite, DMSO, TMAO, thiosulphate and sulphide remains normal, demonstrating its specific role in reducing metals (Beliaev *et al*., 2001). The role of MtrA in reduction of solid Fe(III) (hydr)oxides remains to be determined. Because of its periplasmic location, MtrA is believed to play a critical role in transferring electrons from CymA across the periplasm to the outer membrane proteins such as MtrB (Fig. 1A).

MtrB is not a *c*-Cyt but is nonetheless required for metal reduction (Beliaev and Saffarini, 1998; Lies *et al*., 2005). Based on its amino acid sequence, MtrB is predicted to be a transmembrane protein (Beliaev and Saffarini, 1998). The exact function of MtrB is currently unknown but there is evidence to suggest that it is involved in the proper localization and insertion of MtrC and OmcA into the outer membrane (Myers and Myers, 2002b).

Interestingly, the MtrA polypeptide can be divided into two pentahaem domains, each of which shares sequence similarity with NrfB of *E. coli* (Beliaev and Saffarini, 1998; Clarke *et al*., 2004). Like NrfH (see above), NrfB functions as a physiological electron donor to NrfA (Clarke *et al*., 2004). In addition, heterologously expressed MtrA in *E. coli* is able to serve as an electron donor to the NrfA of *E. coli* (Pitts *et al*., 2003). All these results suggest that *mtrA* most likely evolved from an *nrfB* homologue by gene duplication and fusion (Clarke *et al*., 2004). Given that MtrA and CymA are capable of interacting and carrying out intermolecular ET with NrfA, it is reasonable to speculate that NrfA might function as an ET intermediate between CymA and MtrA during the dissimilatory reduction of solid Fe(III) and Mn(III, IV) (hydr)oxides.

### MtrC and OmcA: two cell surface *c*-Cyts that can serve as terminal reductases towards solid metal (hydr)oxides

Both MtrC and OmcA are lipoproteins that each polypeptide contains 10 haems, and they are located on the extracellullar site of the outer membrane (Table 1) (Myers and Myers, 2003a; 2004). Disruption of the genes encoding OmcA or MtrC does not have any effect on the ability of *S. oneidensis* MR-1 to reduce many soluble electron acceptors, including nitrate and nitrite, but mutants defective in either *mtrC* or *omcA* have reduced rates of MnO_2_ reduction (Beliaev *et al*., 2001; Myers and Myers, 2001; 2002a). Inactivation of *mtrC* lowers the capacity of *S. oneidensis* MR-1 to reduce solid Fe(III) oxides as well (Myers and Myers, 2002a; Lies *et al*., 2005). Likewise, the reduction rate of insoluble silica (Si)-hydrous ferric oxide (HFO) by a Δ*mtrC*/*omcA* double-deletion mutant is only 30% of that of wt (Gorby *et al*., 2006). Overproduction of MtrC can also restore the ability of a Δ*omcA* mutant to reduce MnO_2_ (Myers and Myers, 2003b). This functional redundancy of MtrC and OmcA emphasizes their important roles in dissimilatory reduction of solid Fe(III)/Mn(IV) oxides.

Like CymA, MtrC and OmcA interact with other proteins. MtrC is thought to assist in the proper localization of OmcA to the outer membrane (Myers and Myers, 2001). Recombinant OmcA purified from wt *S. oneidensis* MR-1 cells contains endogenous MtrC. The recombinant MtrC and OmcA that are independently purified from Δ*mtrC*/*omcA* mutant cells form a stable complex *in vitro* with *K*_d_ < 500 nM. Because the stoichiometry of MtrC : OmcA is 1:2, this complex contains at least 30 haems. Each purified protein can reduce Fe(III)-nitrilotriacetic acid (NTA) but, when mixed, their combined activity is greater than the sum of the individual activities, suggesting that MtrC and OmcA interact cooperatively (Shi *et al*., 2006). Consistent with these observations, MtrC and OmcA can be cross-linked *in vivo* (Tang *et al*., 2007).

In addition to soluble Fe(III)-NTA, both purified MtrC and OmcA bind to and reduce crystalline Fe(III) oxide haematite (α-Fe_2_O_3_) (Xiong *et al*., 2006; Lower *et al*., 2007). The turnover numbers of MtrC and OmcA to haematite are 0.26 s^−1^ and 0.11 s^−1^, respectively (Xiong *et al*., 2006; L. Shi, unpubl. data), which are at least three orders of magnitudes slower than those of cytochrome *c* oxidases (COX) to O_2_ (650 s^−1^ for COX of bovine heart and 1230 s^−1^ for COX of *Rhodobacter sphaeroides*) (Han *et al*., 2005; Riegler *et al*., 2005). The extremely low-turnover numbers of MtrC and OmcA towards solid haematite indicate that the ET from proteins to solid minerals might be the rate-limiting step of the ET pathways of *S. oneidensis* MR-1 for dissimilatory reduction of insoluble Fe(III)/Mn(III, IV) (hydr)oxides, although other factors such as potential energy associated with the mineral surfaces may also be important.

Under anaerobic conditions, *S. oneidensis* MR-1 produces extracellular structures that are associated with nano-sized particles of uraninite (UO_2_). MtrC and OmcA, the major components of these structures, might serve as the extracellular terminal reductases to uranyl carbonate complexes, because (i) double deletion of *mtrC*/*omcA* genes considerably lowers the amount of extracellular UO_2_ nano-particles formed, (ii) MtrC and OmcA are colocalized with the UO_2_ nano-particles and (iii) MtrC reduces uranyl carbonate complexes *in vitro* (Marshall *et al*., 2006). Under O_2_-limited conditions, *S. oneidensis* MR-1 cells produce extracellular materials that were termed nanowires, which could conduct a tunnelling current and therefore could be imaged by scanning tunnelling microscopy (STM). Nanowires have been implicated in *S. oneidensis* MR-1-mediated reduction of Si-HFO and generation of electrical current in microbial fuel cells (MFC). MtrC and OmcA were proposed to have a functional role in these materials, as the Δ*mtrC*/*omcA* mutant produces extracellular materials that are not readily imaged by STM and has impaired abilities either to reduce Si-HFO or to generate the current in MFC (Gorby *et al*., 2006). Whether MtrC or OmcA is structural components of nanowires and the properties of nanowires that facilitate electron tunnelling remain to be determined.

Although the mechanism by which MtrC and OmcA are translocated across outer membranes has yet to be determined, the bacterial type II secretion system (T2S) seems to play a critical role. Insertional inactivation of *gspE*, a gene encoding a key component of the T2S, impairs the ability of *Shewanella putrefaciens* strain 200 to reduce solid Fe(III)/Mn(IV) oxides, demonstrating for the first time the direct involvement of T2S in reduction of solid metal oxides (DiChristina *et al*., 2002). Likewise, a *S. oneidensis* MR-1 mutant lacking functional GspD exhibits a similar phenotype to a Δ*mtrC*/*omcA* mutant with respect to the diminished ability to reduce Si-HFO and to generate the current in MFC (Gorby *et al*., 2006). Also functioning as a major component of bacterial T2S, GspD is an outer membrane protein through which certain proteins of Gram-negative bacteria are translocated across the outer membrane into the extracellular space (Fig. 1A). The phenotypic similarity between Δ*mtrC*/*omcA* and insertionally inactivated *gspD* mutants indicates that MtrC and OmcA, which are synthesized in the cytoplasm and matured in the periplasm, might be translocated to the extracellular space through GspD. Consistent with this expectation, examination of *Shewanella* cells cultured under aerobic conditions by atomic force microscopy reveals that their surfaces are relatively smooth. In contrast, cells grown under electron acceptor-limited conditions exhibit the protruding structures or domains, whose height is 18–25 nm with lateral dimension of 35–50 nm, formed on the cell surfaces. Examination with confocal surface-enhanced Raman scattering spectroscopy also suggests that *c*-Cyts are associated with these cell surface domains. Inactivation of *gspD* resulted in the absence of *c*-Cyts on these domains, indicating a critical role played by GspD for translocation of cell surface *c*-Cyts (Biju *et al*., 2007). Our recent comparative proteomic analysis of the proteins released to the growth medium under anaerobic conditions by wt and Δ*gspD* mutant revealed that: (i) MtrC and OmcA were the major *c*-Cyts in the medium, and (ii) their abundances in the medium were significantly reduced by the *gspD* deletion. This result was further substantiated by haem-staining and Western blot analyses (L. Shi, unpubl. data). Thus, in the absence of O_2_ as the terminal electron acceptor, *S. oneidensis* MR-1 cells most likely use the T2S to facilitate the translocation of MtrC and OmcA across the outer membrane to the bacterial surface (Fig. 1A).

Extracellular MtrC and OmcA function as metal reductases with ability to bind and reduce haematite. Because MtrC and OmcA form a high-affinity complex and one of their common functions is to reduce solid metal (hydr)oxides, the extracellular MtrC/OmcA complex should be considered as a single functional unit for metal reduction. Many important questions regarding extracellular MtrC/OmcA complex remain unanswered, such as how it receives the electrons from the periplasm and how it functions at the molecular scale in terms of engaging with and transferring electrons to the substrates. It is clear, however, that *S. oneidensis* MR-1 cells are able to reduce solid metal (hydr)oxides via direct contact between MtrC/OmcA complex and surface of solid metal (hydr)oxides (Fig. 1A).

Formation of nanoscale extracellular structures that contain MtrC/OmcA and appear flexible (Marshall *et al*., 2006; Biju *et al*., 2007) suggests that *S. oneidensis* MR-1 cells might be able to reduce the solid metal (hydr)oxides that are located in regions of sediments and soils where pore sizes limit direct access by the whole cells. The extracellular structures could be extended into small pores where they may contact the (hydr)oxides, consistent with previous results showing that MtrC is involved in the reduction of solid Fe(III) oxide trapped inside porous glass beads (Lies *et al*., 2005).

## Multihaem *c*-Cyts involved in *G. sulfurreducens*-mediated dissimilatory reduction of solid metal (hydr)oxides

All available functional data suggest that multihaem *c*-Cyts play a critical role in mediating the reduction of extracellular metal oxides by *G. sulfurreducens*. When insoluble Mn(IV) oxide is used as the terminal electron acceptor in *G. sulfurreducens* cultures, the two *c*-Cyts OmcE (GSU0618) and OmcS (GSU2504) are identified in culture supernatants after subjecting cells to shear forces generated by blending for 2 min at low speed. Both OmcE and OmcS are predicted to be outer membrane proteins with four and six CX_2_CH motifs respectively (Table 1). Because they are readily released into the solution phase by weak shearing force, OmcE and OmcS seem to be loosely associated with the cell surface. Deletion of either gene decreases the ability of *G. sulfurreducens* to reduce Mn(IV) as well as Fe(III) oxides. A gene located immediately downstream of *omcS*, *omcT* (GSU2503), also encodes a hexahaem *c*-Cyt that is 62% identical to OmcS (Table 1). The *omcS* gene is either transcribed by itself or co-transcribed with *omcT*, while *omcT* is only co-transcribed with *omcS*. Deletion of *omcT* affects the expression of *omcS* and lowers the rates of Mn(IV) and Fe(III) oxides reduction by *G. sulfurreducens*. Deletion of *omcE*, *omcS* or *omcT*, however, does not have any effect on the ability of *G. sulfurreducens* to reduce soluble Fe(III)-citrate. These results indicate that OmcE, OmcS and OmcT are likely to be directly involved in reduction of insoluble Fe(III)/Mn(IV) oxides by *G. sulfurreducens* (Mehta *et al*., 2005).

In contrast to *S. oneidensis* MR-1 cells that mainly use *c*-Cyts for ET to solid metal (hydr)oxides, it has been suggested that *G. sulfurreducens* uses T4P to transfer the electrons directly from the outer membrane to extracellular metal (hydr)oxides (Reguera *et al*., 2005; Lovley, 2006) (Fig. 1B). Because T4P are anchored to the inner membrane and extend across the periplasm and outer membrane into the extracellular space, the pilus of *G. sulfurreducens* might be able to receive electrons from the proteins associated with the inner membrane, periplasm and/or outer membrane. Therefore, OmcE and OmcS might transfer electrons to pili (Mehta *et al*., 2005). Like the model itself, however, no biochemical or biophysical data are available to substantiate this speculation.

## Conceptual models and knowledge gaps

Because of their abilities to transfer electrons rapidly, to catalyse chemical reactions and their appropriate electrochemical properties, multihaem *c*-Cyts are the key structural components employed by both *S. oneidensis* MR-1 and *G. sulfurreducens* for transferring electrons accumulated at the inner membrane during respiration to extracellular terminal electron acceptors when soluble electron acceptors are absent. However, the proposed roles played by these multihaem *c*-Cyts in *S. oneidensis* MR-1 differ significantly from those in *G. sulfurreducens*.

As already discussed (see above), multihaem *c*-Cyts CymA, MtrA, MtrC and OmcA in *S. oneidensis* MR-1 all play leading roles in facilitating ET from the respiratory chain in the inner membrane, through the periplasm (i.e. CymA→MtrA) and, finally to the outer membrane protein complex MtrC/OmcA facing the extracellular space, which are capable of transferring electrons directly to solid metal (hydr)oxides (Fig. 1A). Indeed, MtrC and OmcA have been purified, reconstituted and demonstrated to function as terminal reductases of haematite. MtrB does not appear to contain any haems but might function as an organizing centre that facilitates ET from MtrA at the periplasm to the MtrC/OmcA at cell surface.

In contrast to our understanding of metal reduction in *S. oneidensis* MR-1, much less is known about the mechanisms underlying metal reduction in *G. sulfurreducens*. The multihaem *c*-Cyts OmcE and OmcS might play supporting roles to facilitate ET to the T4P structural complex, which then relays electrons directly to the surface of solid metal (hydr)oxides (Fig. 1B). This suggestion represents a new ET mechanism not previously observed in nature, as the pilin proteins in this structure lack obvious haem or other metal binding sites. Indeed, this model ‘contrasts with the nearly universal concept that outer-membrane cytochromes are the proteins that transfer electrons to Fe(III) oxides’ (Reguera *et al*., 2005). An important prediction is that the structural pilin proteins in the T4P complex can receive and transport electrons. However, isolation and characterization of these proteins need to be carried out to test whether they have the electrochemical properties that are consistent with this model. An alternative hypothesis is that, like *S. oneidensis* MR-1, *G. sulfurreducens* uses multihaem *c*-Cyts to mediate direct ET to solid metals, taking advantage of the nearly universal structural motifs commonly used in biological systems to mediate ET reactions. In this respect, it is interesting to note that the T2S and T4P biogenesis machines in *Shewanella* and *Geobacter*, respectively, evolved from a common ancestor (Filloux, 2004). Their involvement in *S. oneidensis* MR-1- and *G. sulfurreducens*-mediated dissimilatory reduction of solid metal (hydr)oxides, respectively, demonstrates a common biological function shared by these two systems. Furthermore, nearly all key structural components of the ET pathway used by *S. oneidensis* MR-1 to reduce solid metal (hydr)oxides have their putative counterparts in *G. sulfurreducens* (Lovley, 2006) and one of the T2Ss in *G. sulfurreducens* is required for reducing solid Fe(III)/Mn(IV) oxides (Mehta *et al*., 2006), suggesting that the general strategy for ET to solid metal (hydr)oxides might be well conserved in *Shewanella* and *Geobacter*.

Despite recent advances in understanding the roles of the multihaem *c*-Cyts in *S. oneidensis* MR-1-mediated dissimilatory reduction of solid metal (hydr)oxides, four important questions must be answered to provide a basic framework for understanding of the physiology of extracellular metal (hydr)oxide reduction by DMRB. Our primary question is how the T2S facilitates translocation of MtrC/OmcA across the outer membrane. To date, the pullulanase of *Klebsiella oxytoca* is probably the only outer membrane lipoprotein whose translocation via the T2S is well characterized (Francetic and Pugsley, 2005). If confirmed, MtrC/OmcA would provide another model system to investigate how lipoproteins are translocated across the outer membrane by T2S. A mechanistic understanding of the MtrC/OmcA translocation process will be the first step to unravel the molecular mechanisms that control the proper placement of MtrC/OmcA where they can directly contact solids outside the cells. Second, which domain elements in the MtrC/OmcA complex are associated with binding and reduction of solid metal (hydr)oxides? These proteins were the first isolated proteins for which the ability to bind and reduce solid metal (hydr)oxides was directly demonstrated. An understanding of their mechanisms has great potential both in understanding bacterial physiology and with respect to practical applications involving, for example, the construction of biological fuel cells whose greater efficiency will require the development of stable and long-lived protein-based materials. Third, the role of MtrB in promoting ET to the cell surface remains uncertain, and structure/function measurements are necessary to reveal the mechanisms by which MtrB facilitates, directly or indirectly, ET. Such investigations will need to identify any possible interactions between MtrB and the multihaem *c*-Cyts involved in the process of reduction of solid metal (hydr)oxides. This is critical, as MtrB is essential for *S. oneidensis* MR-1 to reduce the solid metal (hydr)oxides. Finally, it is unclear how the electrons are transferred from CymA to MtrA. Does CymA transfer electrons directly to MtrA or require NrfA and/or other periplasmic proteins as ET intermediates? Answers for these questions are critical to our understanding of how electrons are transferred across the periplasm.

In summary, although considerable gaps in our knowledge about the bacterial ET pathways used for dissimilatory reduction of solid metal (hydr)oxides remain, the common feature of these pathways found in *S. oneidensis* MR-1 and *G. sulfurreducens* is that multihaem *c*-Cyts, which are strategically located at the inner membrane, periplasm and outer membrane and are able to interact with other proteins, facilitate ET across the physical barrier of bacterial cell envelope to the surface of solid metal (hydr)oxides.
